# Brief Report: The Effectiveness of Hugging a Huggable Device Before Having a Conversation with an Unfamiliar Person for Autism Spectrum Disorders

**DOI:** 10.1007/s10803-021-05173-8

**Published:** 2021-07-22

**Authors:** Hirokazu Kumazaki, Hidenobu Sumioka, Taro Muramatsu, Yuichiro Yoshikawa, Jiro Shimaya, Ryoichiro Iwanaga, Hiroshi Ishiguro, Tomiki Sumiyoshi, Masaru Mimura

**Affiliations:** 1 Department of Preventive Intervention for Psychiatric Disorders, National Institute of Mental Health, National Center of Neurology and Psychiatry, 4-1-1. Ogawahigashimachi, Kodaira, Tokyo, 187-8553 Japan; 2 Department of Neuropsychiatry, Keio University School of Medicine, Tokyo, Japan; 3 Hiroshi Ishiguro Laboratories, Advanced Telecommunications Research Institute International, Kyoto, Japan; 4 Department of Systems Innovation, Graduate School of Engineering Science, Osaka University, Osaka, Japan; 5 Department of Occupational Therapy, Graduate School of Health Sciences, Nagasaki University, Nagasaki, Japan

**Keywords:** Autism spectrum disorders, Robot, Sensory over-responsivity, Sensory seeking, Tactile, Social anxiety

## Abstract

Sensory overresponsivity (SOR) emerges before anxiety and positively predicts subsequent increasing levels of anxiety in individuals with autism spectrum disorder (ASD). Sensory seeking behavior occurs as compensation for SOR, and individuals may seek sensory input in one sensory domain to compensate for SOR. Tactile seeking behavior is sufficient to decrease social anxiety in communicating with unfamiliar people. We assessed the effectiveness of hugging a huggable device before a conversation for reducing the psychological stress associated with speaking to an unfamiliar person or robot. Our analysis showed a significant effect, with *Hugvie* contributing to decreased stress for both interlocutors. Thus, this study demonstrated the efficacy of hugging it before conversation, which emphasizes the importance of tactile seeking for individuals with ASD.

**Supplementary Information:**

The online version contains supplementary material available at 10.1007/s10803-021-05173-8.

## Introduction

Autism spectrum disorder (ASD) is a developmental disability that can cause significant social, communication, and behavioral challenges. Interpreting the facial expressions of others, being aware of one’s own body language as projected to others, and sensory overload issues caused by the speaking environment may be challenges for autistic individuals (Kapp, 2013). In particular, speaking to unfamiliar people is one of the core areas of difficulty for autistic individuals partly because they have a lower imagination compared to a typical control. They tend to feel social anxiety when they have to speak to unfamiliar people (Sung et al., 2011) and tend to withdraw from these opportunities.

Interventions such as cognitive behavioral therapy (CBT) and pharmacotherapy have been used to reduce social anxiety when speaking to unfamiliar people in the general population (British Psychological Society, 2013). However, previous studies suggest that autistic individuals may attain less favorable outcomes from CBT due to the impact of cooccurring anxiety symptoms (Maddox et al., 2016; Pellecchia et al., 2015; Spain et al., 2017). Pharmacotherapy such as anxiolytics also seems to be less effective overall in these patients than in the general population (Guenné et al., 2020). Therefore, the development of alternative treatments for social anxiety in autistic individuals is desired.

**Fig. 1:**
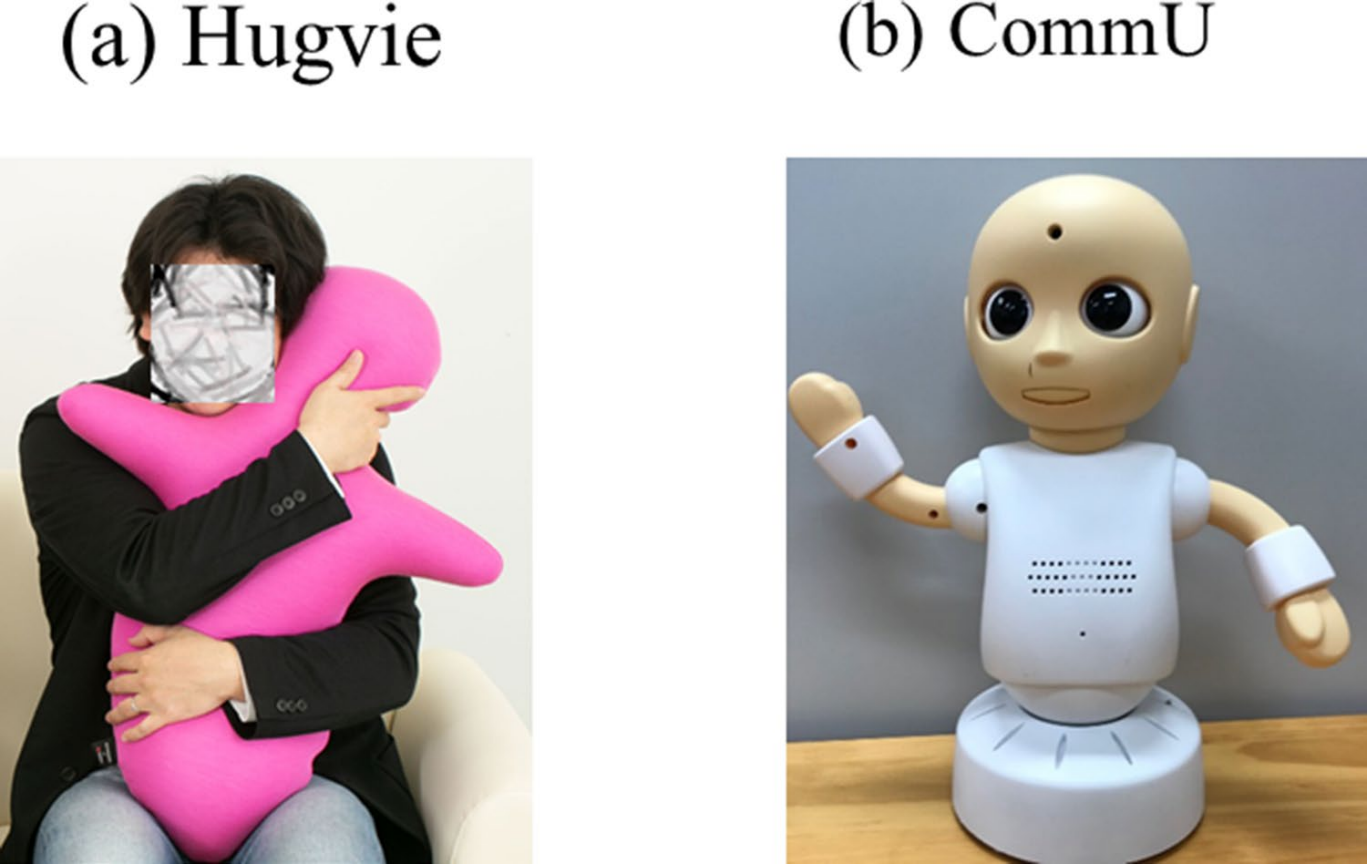
**a** *Hugvie*, **b** CommU

There is a strong, positive association between anxiety and sensory overresponsivity (SOR) in autistic individuals (Ludlow et al., 2015). It has been observed that SOR emerges before anxiety and positively predicts subsequent increasing levels of anxiety (Green et al., 2012). Sensory seeking behavior including tactile seeking behavior is an attempt to self-regulate anxiety (Miller et al., 2007). Autistic individuals seek sensory input in one sensory domain to decrease anxiety, which is associated with the prevention of SOR (Schoen et al., 2014). A previous study suggests that hugging “*Hugvie*” (Fig. 1), which is a human-shaped cushion (75 cm high and 600 g) that provides an outlet for participants' tactile seeking behavior, is effective at decreasing stress in the moment in the general population (Sumioka et al., 2013). Given that hugging “*Hugvie*” is tactile seeking behavior, we hypothesized that hugging “*Hugvie*” before communication would be sufficient to decrease social anxiety in autistic individuals when communicating with unfamiliar persons.

The primary aim of this study is to examine whether physical contact hugging *Hugvie* before a conversation can reduce the psychological stress associated with speaking to unfamiliar people. In the current work, we assessed electrodermal activity (EDA), which is a reliable biomarker of psychological stress and is heavily linked to social anxiety, during communication with unfamiliar persons after resting and hugging *Hugvie in a sitting position* or after resting as usual in a sitting position. In addition, because current assessment for ASD is somewhat unreliable and insensitive (Frith, 2012), recent work has suggested the importance of measuring both physiological arousal level and self-reported scores (Kumazaki et al., 2017; Mikita et al., 2015; Simon & Corbett, 2013) in autistic individuals to improve the accuracy of evaluation. Therefore, we sought to assess both EDA and self-reports to obtain a more objective view of the psychological stress on the participants.

Furthermore, the existing literature on the social communication outcomes of interventions for ASD depends upon human interlocutors. There is a need for quantitative, objective measurements of social functioning for autistic individuals. Evaluation measures that involve direct observations of the participant’s interactions are affected by the compatibility between the interlocutors and the participant, and the interlocutors are unable to accurately repeat the same actions from one session to another (Lord et al, 2012). Therefore, the need for a more objective assessment tool is imperative. Structured interactions with robots could create standardized social situations to elicit particular social behaviors. In fact, previous studies using robot (Anzalone, 2014, 2019) make comparison of the responses of different participants in standardized situations and different response of the same participant across time possible. Using humanoid robots as interlocutors could offer a replicable measurement system. In this study, we used not only unfamiliar persons but also unfamiliar humanoid robots as interlocutors.

## Methods

### Participants

The participants were autistic adolescents and young adults recruited from our institute, which is well known in Japan for specializing in developmental disorders and related conditions. All procedures involving human participants were conducted in accordance with the ethical standards of the institutional and/or national research committee and the 1964 Declaration of Helsinki and its later amendments or comparable ethical standards. After receiving a complete explanation of the study, all participants and their guardians agreed to participate. All participants provided written informed consent. The inclusion criteria for the participants were as follows: (1) age of 15–24 years, (2) male, and (3) IQ ≥ 70. The participants were diagnosed by psychiatrists with more than 15 years of experience in ASD using the criteria in the Diagnostic and Statistical Manual of Mental Disorders (DSM-5) (American Psychiatric Association, 2013) and standardized criteria taken from the Diagnostic Interview for Social and Communication Disorders (DISCO) (Leekam et al., 2002) at the time of enrollment in the study. The DISCO is reported to have good psychometric properties. It also contains items on early development and a section on activities of daily life, thereby giving the interviewer an idea of the individual’s level of functioning in several areas rather than for only social functioning and communication (Wing et al., 2002). All participants who were diagnosed with childhood autism, atypical autism or Asperger’s syndrome according to the DISCO were included in this study. To exclude other psychiatric diagnoses, we implemented the Mini-International Neuropsychiatric Interview (M.I.N.I.) (Sheehan et al., 1998) and found that none of the participants had any other psychiatric disorders.

All participants completed the Autism Spectrum Quotient-Japanese version (AQ-J) (Wakabayashi et al., 2004), which was used to evaluate ASD-specific behaviors and symptoms. The AQ-J is a short questionnaire with five subscales (i.e., social skills, attention switching, attention to detail, imagination, and communication). Previous studies using the AQ-J have been replicated across cultures (Wakabayashi et al., 2007). The AQ is also sensitive to the broader autism phenotype (Wheelwright et al., 2010). IQ was measured using the Wechsler Adult Intelligence Scale-Fourth Edition (Wechsler, 2008).

The severity of social anxiety symptoms was measured using the Liebowitz Social Anxiety Scale (LSAS) (Liebowitz, 1987). This clinician-administered scale consists of 24 items, including 13 items that describe performance situations and 11 items that describe social interaction situations. Each item is separately rated for “fear” and “avoidance” using a 4-point categorical scale. According to receiver operating curve analyses, an LSAS score of 30 is correlated with minimal symptoms and is the best cutoff value for distinguishing individuals with social anxiety disorder from those without (Mennin et al., 2002).

All participants also completed the Adolescent/Adult Sensory Profile (AASP; Brown & Dunn, 2002), which is a self-report questionnaire of sensory processing for individuals aged 11 years and older. The internal consistency coefficients of the AASP range from 0.64 to 0.78 for the quadrant scores. In this study, before the experiment, the participants indicated how often they exhibited certain behaviors related to sensory experiences using a one-through-five scale, ranging from “almost never” (score of 1) to “almost always” (score of 5). The AASP examines four different “quadrants” of sensory processing: low registration, sensation seeking, sensory sensitivity, and sensation avoidance.

### Procedures

**Fig. 2:**
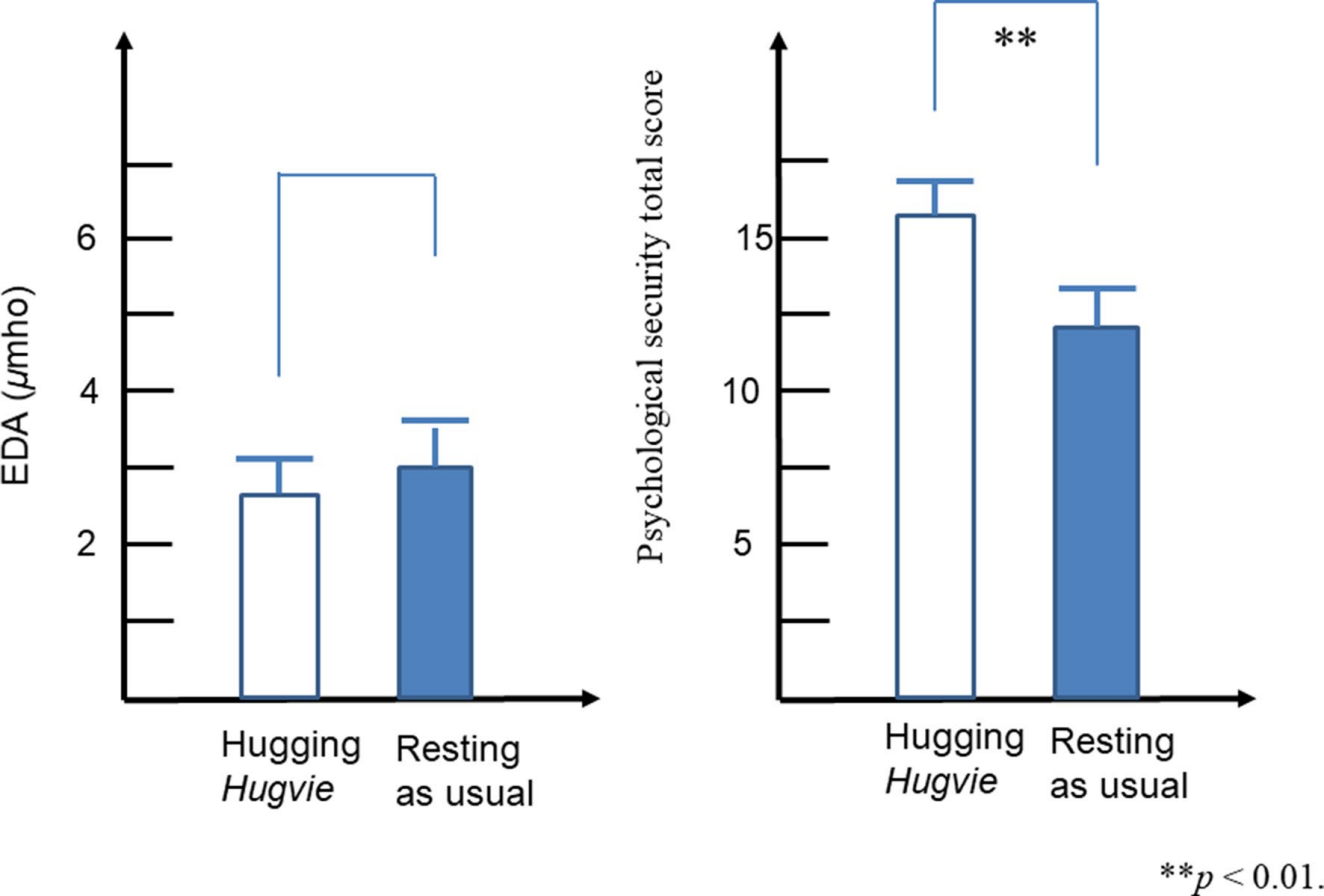
Differences of the EDA and psychological security total score in the conversations with an unfamiliar person between hugging *Hugvie* and resting as usual condition

In the present study, the participants had a conversation for approximately five minutes with an unfamiliar person or humanoid robot “CommU” (Fig. 2) (Kumazaki, 2018; Shimaya et al., 2016), which was controlled by an unfamiliar person. Before the conversation, the participants rested in the waiting room for approximately five minutes while hugging a *Hugvie* or rested as usual. Ten participants participated in this study. All the participants experienced four sessions (i.e., a conversation with an unfamiliar person after resting while hugging *Hugvie* in a sitting position, a conversation with CommU after resting while hugging *Hugvie* in a sitting position, a conversation with an unfamiliar person after resting as usual in a sitting position, and a conversation with CommU after resting as usual in a sitting position). The order of the sessions was randomly assigned to each participant. We used the same unfamiliar people and robots in each conversation. There were 5-min intervals between each session. Conversations were conducted based on prepared scripts.

During conversation sessions, the scripts were varied slightly to promote engagement but followed the same basic structure (see supplementary material for examples of the scripts). The interlocutor did not know whether the participants hugged *Hugvie* before the conversation. To reduce sequence effects, the conversation (i.e., human or CommU) and resting (hugging *Hugvie* or as usual) orders were randomized.

During the conversation, the participants wore an E4 wristband. After each conversation, all participants completed questionnaires that were scored using Likert-rating scales regarding ease with talking, fun talking, confidence talking, and amused while talking. The ratings ranged from 1 (not at all) to 5 (very). We calculated the Cronbach’s alpha reliability coefficient for 4 items (i.e., ease with talking, fun talking, confidence talking, and amused while talking) of each self-reported score established in a given condition to be 0.823. Hence, we computed a single composite score (i.e., psychological security total score) by adding the rate of each self-reported score.

### E4-wristband

The E4 wristband (Empatica Co) is a wearable device that has been used in many clinical trials and in medical research for a variety of purposes (McCarthy et al., 2016). The E4 wristband is equipped with a collection of sensors that collect physiological data such as EDA in real time. Using the E4-wristband is easy and convenient. Users can wear it like a watch on their wrist. Data recording starts once the button on the E4 is pressed. The temporal resolution is 0.2 s in streaming mode, which gives us high-precision data measurement with respect to time. Participants sported the E4 wristband on the non-dominant hand. Python 3.7 was used to analyze the data. We discarded the motion artifact based on the sensor values of the 3-axis accelerometer inside the E4-wristband. None of the participants engage in problem behavior during the conversation or rest periods.

### Humanoid Robot

In this study, we used the humanoid robot “CommU” (Fig. 1; Vstone Co., Ltd.) (Kumazaki et al., 2018; Shimaya et al., 2016), which is 304 mm tall and has 14 degrees of freedom: waist (2), left shoulder (2), right shoulder (2), neck (3), eyes (3), eyelids (1), and lips (1). It has clearly distinguishable eyes, which are its most distinct and prominent feature. “CommU” is capable of shifting its gaze and blinking, and by the smooth movement and positioning of its eyelids, it can demonstrate a range of simplified expressions that are less complex than those of a real human face. Its child-like shape is considered to be easy to anthropomorphize and help prevent fear among interlocutors. A commercial software (AITalk, AI Co. Ltd) is used to synthesize its voice from input text, which sounds like a flat voice rather than one with humanlike intonation. “CommU” makes very little noise, and the noise it generates is not distressing to interlocutors.

### Hugging Devices

We used a human-shaped cushion called *Hugvie*, manufactured by Nishikawa Co., (75 cm high and 600 g) that was designed as a target for tactile sensory seeking behavior. *Hugvie* is a soft cushion filled with polystyrene microbeads and covered with mixed fiber that contains acrylic and rayon. It resembles a person opening its arms for a hug, enabling a hug experience.

### Statistical Analysis

We performed the statistical analyses using SPSS version 27.0 (IBM, Armonk, NY, USA). The EDA during the conversation with unfamiliar persons and total score of each self-reported scores (i.e., ease with talking, fun talking, confidence talking, and amused while talking) after the conversation were compared between the hugging *Hugvie* condition and the resting as usual condition using a paired t-test. The EDA during the conversation with CommU and total score of each self-reported score (i.e., ease with talking, fun talking, confidence talking, and amused while talking) after the conversation were also compared between the hugging *Hugvie* condition and the resting as usual condition by using a paired t-test. We have conducted post-hoc power analyses for the EDA during the conversation with unfamiliar persons and the psychological security total score after the conversation. In addition, we have conducted post-hoc power analyses for the EDA during the conversation with CommU and psychological security total score after the conversation. We have compared the number of letters used in the verbal output during the conversation with unfamiliar persons between after resting while hugging *Hugvie* and the resting as usual condition using a Wilcoxon signed-rank test. In addition, we have compared the number of letters used in the verbal output during the conversation with CommU between after resting while hugging *Hugvie* and the resting as usual condition using a Wilcoxon signed-rank test.

Pearson product-moment correlation coefficients were used to explore the relationships between the total score of each self-reported score (i.e., psychological security total score) and the EDA measures collected within a given condition, AQ scores, quadrant scores on the AASP, LSAS scores, and VIQ score. We employed an alpha level of 0.05 for these analyses. We calculated the effect size (Cohen’s d) of the EDA during the conversation with unfamiliar persons and psychological security total score after the conversation between the hugging *Hugvie* condition and the resting as usual condition by dividing the difference in the mean score between the *Hugvie* condition and the resting as usual condition using the pooled standard deviation of the score. We also calculated the effect size (Cohen’s d) of the EDA during the conversation with unfamiliar CommU and psychological security total score after the conversation between the hugging *Hugvie* condition and the resting as usual condition by dividing the difference in the mean score between the *Hugvie* condition and the resting as usual condition using the pooled standard deviation of the score. According to Cohen (1988), the values of 0.2, 0.5, and 0.8 indicate small, moderate, and large effect sizes respectively.

## Results

### Demographic Data

In total, ten autistic individuals participated in the study. All participants completed the experimental procedure and the questionnaires. Five participants had unusually high scores in the sensory avoidance and three showed sensory sensitivity. Based on the LSAS scores, five participants had social anxiety. The details are presented in Table 1.

**Table 1:** Descriptive statistics of participants (n = 10)

Characteristics	M (SD)	Range
Age in years	21.3 (2.4)	19–25
Full-scale IQ	90.3 (13.6)	72–117
Performance IQ	95.4 (18.2)	71–124
Verbal IQ	87.2 (13.6)	71–108
AQ-J	33.1 (4.6)	26–38
LSAS	46.0 (9.3)	35–63
AASP		
Low Registration	33.6 (9.4)	18–49
Sensation Seeking	38.3 (11.9)	24–62
Sensory Sensitivity	34.4 (11.7)	17–51
Sensation Avoiding	33.4 (8.0)	19–46

*M* mean, *SD* standard deviation, *AQ-J* autism spectrum quotient, Japanese version. In the AQ-J, higher scores reflect a greater number of ASD-specific behaviors

*LSAS* Liebowitz Social Anxiety Scale, *AASP* Adolescent/Adult Sensory Profile

### Main Result

**Fig. 3:**
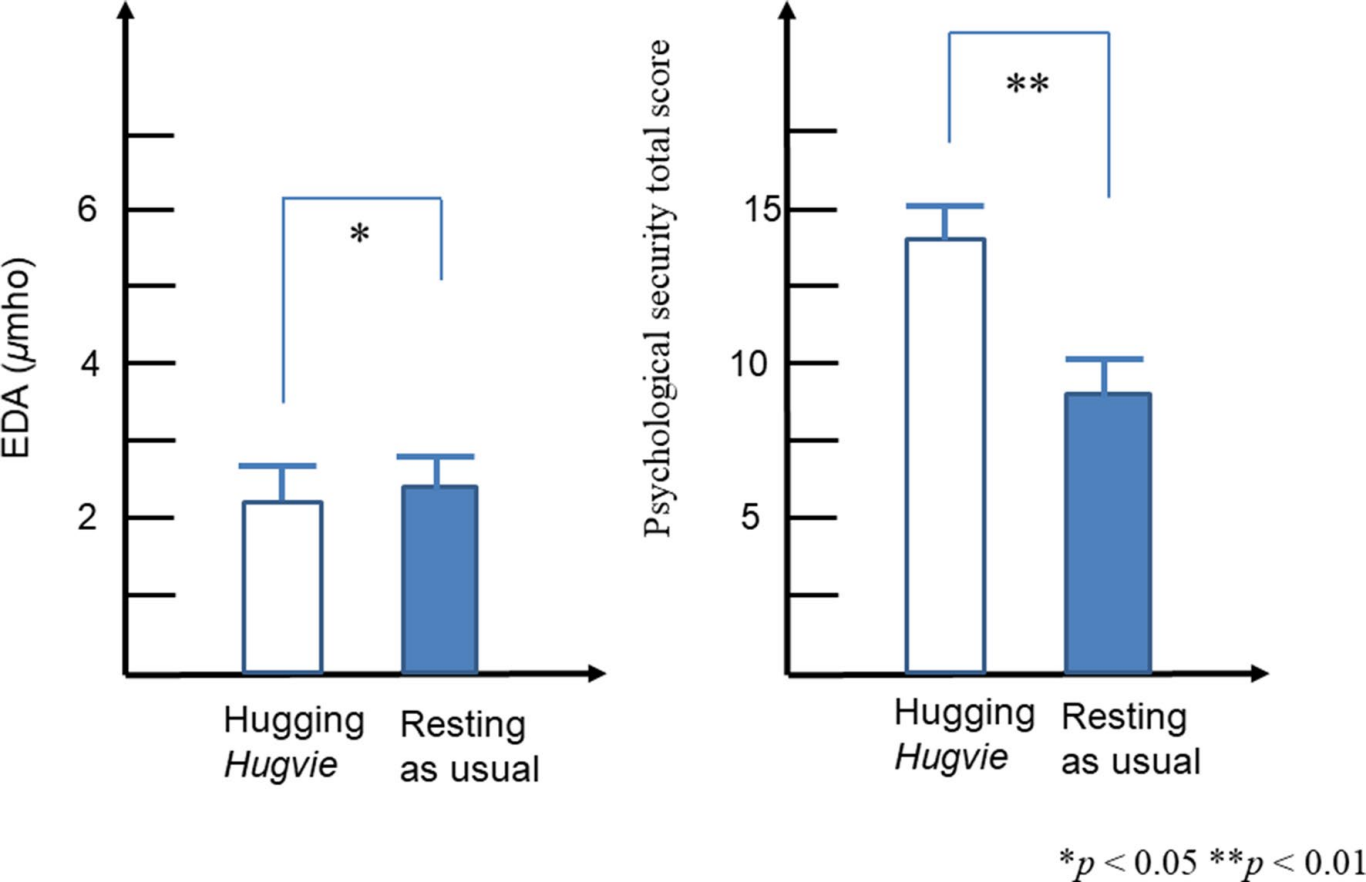
Differences of the EDA and psychological security total score in the conversations with CommU between hugging *Hugvie* and resting as usual condition

There were statistically significant differences in the conversations with an unfamiliar person between after hugging *Hugvie* and after rest as usual for the psychological security total score (*p* = 0.001). There were marginally significant differences in the conversations with an unfamiliar person between hugging *Hugvie* and after rest as usual for the EDA (*p* = 0.093). There were statistically significant differences in the conversations with CommU between the hugging *Hugvie* condition and the rest as usual condition for the EDA (*p* = 0.047) and the psychological security total score (*p* = 0.001). Details are presented in Table 2 and Figs. 2 and 3.

**Table 2:** Means and standard error of the mean for EDA during the conversation and self-reported scores (i.e., ease with talking, fun talking, nervousness while talking, boredom while talking) after the conversation with an unfamiliar person and commu between the hugging hugvie condition and the resting as usual condition

	Hugging Hugvie (M, SEM)	Resting as usual (M, SEM)	Statistics			
			*t*	*df*	*p*	*ES(d)*
Conversation with unfamiliar person						
EDA (*µ*mho)	2.63 (1.55)	3.02 (1.72)	− 1.878	9	0.093	0.64
Self-reported score						
Ease with talking	4.30 (0.26)	2.60 (0.40)				
Fun talking	3.70 (0.40)	2.60 (0.27)				
Confidence with talking	2.90 (0.38)	1.80 (0.20)				
Amused while talking	2.90 (0.43)	1.90 (0.23)				
Psychological Stress Total score	15.30 (1.34)	11.60 (2.17)	4.772	9	0.001**	0.82
Conversation with CommU						
EDA (*µ*mho)	2.36(1.64)	2.66 (1.67)	-2.294	9	0.047*	0.79
ease with talking	4.10 (0.23)	3.10 (0.41)				
fun talking	3.70 (0.21)	2.90 (0.41)				
confidence with talking	3.70 (0.34)	2.60 (0.43)				
amused while talking	3.80 (0.36)	3.00 (0.42)				
Psychological Stress Total score	14.20 (2.25)	9.70 (2.36)	4.590	9	0.001**	0.65

*M*: mean; SEM: standard error of the mean

*p < 0.05

**p < 0.01

*EDA* Electrodermal activity, *ES* Effect size

There were marginally significant differences in the number of letters used in the verbal output during the conversation with unfamiliar persons between after resting while hugging *Hugvie* (228.2 ± 69.2) and the resting as usual condition (205.3 ± 74.0) (*p* = 0.094). There were marginally significant differences in the number of letters used in the verbal output during the conversation with CommU between after resting while hugging *Hugvie* (273.4 ± 82.0) and the resting as usual condition (250.5 ± 69.8) (*p* = 0.090).

There were no significant relationship between the psychological security total score and the EDA measures in the conversation with unfamiliar Person for the hugging *Hugvie* condition (*p* = 0.078,γ = 0.581) and the resting as usual condition (*p* = 0.135, γ = 0.507), and in the conversation with CommU for the hugging *Hugvie* condition (*p* = 0.208, γ = 0.436) and the resting as usual condition (*p* = 0.251, γ = 0.401). There was a significant relationship between the sensation seeking subscores of the AASP and the EDA measures in the conversation with the unfamiliar person for the hugging *Hugvie* condition (*p* = 0.037, γ = 0.662), and CommU for the hugging *Hugvie* condition (*p* = 0.046, γ = 0.641). We could not find other significant relationship between the psychological security total score and the EDA measures collected within a given condition, AQ scores, quadrant scores on the AASP, LSAS scores, and VIQ score.

## Discussion

In the current study, we assessed the effectiveness of hugging a huggable device before having a conversation for reducing psychological stress while speaking to an unfamiliar person or robot. Our analysis showed a significant effect, with *Hugvie* contributing to decreased stress for both interlocutors. Thus, this study demonstrated the efficacy of hugging a *Hugvie* before having a conversation, which emphasizes the importance of tactile seeking for autistic individuals.

Autistic adolescents and young adults are able to self-report psychiatric symptoms (Hurtig et al., 2009). They may be more accurate reporters of their own mood dysregulation than their caregivers (Schupp et al., 2013). Therefore, the results of the self-report questionnaire, which measured ease with talking, fun talking, confidence talking, and amused while talking to an unfamiliar person or robot after hugging *Hugvie* and after resting as usual, are reliable.

In this study, we also used the physiological measure of EDA levels to obtain a more objective view of psychological stress in autistic individuals. Significant differences in EDA were observed during each conversation type (i.e., unfamiliar person and CommU) between the hugging *Hugvie* and resting as usual conditions. Given that measurement of EDA has been shown to be well tolerated in autistic individuals and is sensitive to changes in arousal and emotional states in this population (Ferguson et al., 2017; O'Haire et al., 2015; Schupak et al., 2016; Zamzow et al., 2016), the EDA results indicate that hugging a *Hugvie* before having a conversation reduces psychological stress during a conversation for both interlocutors.

The effect of using *Hugvie* on the EDA was only significant in the case of using robots for interlocutors. Autistic individuals often achieve a higher degree of task engagement through the interaction with robots than through the interaction with human trainees. Specifically, their task engagement is better when facing a robot than when facing a human (Kumazaki, 2020). Hence, it is possible that, by using robots for interlocutors, their engagement gets better; therefore, the effect of using *Hugvie* on EDA becomes significant.

In this study, we used not only unfamiliar persons but also the humanoid robot “CommU” as interlocutors. Robots can repeat given behaviors exactly. Robots allow participants to control and replicate a smooth and accurate conversation, regardless of reactions, contributing to a more structured and standardized intervention (Feil-Seifer & Matarić, 2009; Scassellati, 2007). Owing to their high reliability and high reproducibility, the results could generalize to other similar situations.

The results of this preliminary efficacy study demonstrated that simply hugging a huggable device before having a conversation contributed to self-reported increases in ease with talking, fun talking, confidence talking, and amused while talking and, importantly, to corresponding reductions in biological indicators of psychological stress. In addition, there was a significant relationship between the extent of sensation seeking behavior and the EDA, which is a reliable biomarker of psychological stress. Previous work suggests that electrodermal variability correlated with the ASD symptom severity (Fenning et al., 2017). However, there was no significant relationship between the EDA and AQ in this study. Future study with a larger sample may clarify the relationship. Autistic individuals may have the mirror neurons deficit and could be weak in empathetic social interaction (Rustichini et al., 2008). By hugging *Hugvie*, their empathy increases, and they could possibly reduce the psychological stress associated with speaking to an unfamiliar person or robot. Interestingly, in this study, the participants were unfamiliar with *Hugvie* before the experiment. Some autistic individuals require more time to get accustomed to textures (Mikkelsen et al., 2018; Puts et al., 2014; Tommerdahl et al., 2007). In the case of these autistic individuals, if they become accustomed to huggable devices, further effects can be expected. To confirm the effect, we should investigate the impact of long-term use of *Hugvie* on autistic individuals in future work.

Previous studies have reported that the perception of touch differs between autistic individuals and those with typical development (Cascio et al., 2008; Hilton et al., 2007; Lane et al., 2010; Tommerdahl et al., 2007). It is presumed that autistic individuals generally judge textures to be more pleasant than controls (Cascio et al., 2008). Given these factors, it is difficult to conclude whether the results of this study (i.e., hugging a huggable device before having a conversation can reduce the psychological stress associated with speaking to an unfamiliar person and robot) can be applied to the general population and other clinical populations that struggle with social interactions or sensory processing issues.

We acknowledge several limitations of our study. The first is the relatively small number of participants. The power for the EDA during the conversation with unfamiliar persons and psychological security total score after the conversation compared between the hugging *Hugvie* condition and the resting as usual condition by using a paired t-test were 0.44 and 0.63, respectively. The power for the EDA during the conversation with CommU and psychological security total score after the conversation compared between the hugging *Hugvie* condition and the resting as usual condition using a paired t-test were 0.60 and 0.45, respectively. Our post-hoc power analysis revealed that approximately 22 samples for EDA and 14 samples for psychological security total score in the unfamiliar person condition and approximately 15 samples for EDA and 21 samples for psychological security total score in the CommU condition would be required to obtain statistical power at the recommended 0.80 level. Larger sample sizes are necessary to provide more meaningful self-report results and EDA data. In addition, all participants were male. Although there is a strong male bias documented among autistic individuals, given the sex differences in sensory symptoms in autistic individuals (Kumazaki et al., 2015; Lai et al., 2011), future research should include female participants. We have excluded autistic individuals whose IQs were lesser than 70, since they possibly would not be able to understand the content of the research. The result of this study cannot be generalized to autistic individuals who have IQs < 70. Another limitation is the comparatively short interaction between the participants and the robot; however, 10 min per session might be appropriate for the specific characteristics of autistic individuals, and all participants completed the trial. In this study, we did not assess the measures of both the EDA and the self-rated acute stress in baseline and post-conversation. Future studies assessing the measures of both the EDA and the self-rated acute stress in baseline and post-conversation generate more meaningful data. We did not assess sensory processing based on direct observation. Additional sensory processing data based on direct observation would have ensured a more complete characterization of the participants' unique sensory processing styles. Ferguson et al. (2019) reported that 60% of individuals with severe ASD showed an anticipatory rise in the EDA in the 5 min prior to onset of problem behavior, and that after a problem behavior the EDA only returned to baseline levels (over an average observation period lasting 20 min) in 45% of cases. These findings highlight the importance of accounting problem behavior, and careful measurement of the baseline and recovery in EDA studies of this kind. Although participants in the present study did not engage in problem behaviors, the 5-min periods that separated conversations may not have always been sufficient for EDA to return to baseline. Future studies with long periods, which are sufficient for return of the EDA to baseline are needed. Finally, only autistic individuals were included. To more clearly elucidate the effect of hugging *Hugvie* before a conversation, it is important to study individuals without ASD and compare their data to those of autistic individuals.

To the best of our knowledge, this study is the first to evaluate the effect of hugging a huggable device before having a conversation. Hugging a huggable device appears to be useful for decreasing the psychological stress associated with having a conversation in autistic individuals. *Hugvie* may be considered an appropriate tool for autistic individuals to overcome speaking to unfamiliar people. To draw definitive conclusions regarding the efficacy of hugging the *Hugvie*, these promising results warrant further studies with larger, more diverse samples of autistic individuals and that use a longitudinal design. In addition, in the near future, neuropsychological studies to examine the difference of nature in stimuli between ASD patients and non-ASD participants are needed. Furthermore, neuroimaging studies to examine the meaning and therapeutic feasibility of efficient stimuli for ASD patients are needed.

## Supplementary Information

Below is the link to the electronic supplementary material.

Supplementary file1 (DOCX 32 kb)
