# Supplementary material for: Brief Report: The Effectiveness of Hugging a Huggable Device Before Having a Conversation with an Unfamiliar Person for Autism Spectrum Disorders
Source: J Autism Dev Disord. 2021 Jul 22;52(7):3294–303. doi: 10.1007/s10803-021-05173-8 (PMC9213352; doi:10.1007/s10803-021-05173-8)
Supplement: Supplementary file 1 — Supplementary file1 (DOCX 32 kb) [file 10803_2021_5173_MOESM1_ESM.docx]

**Sample scripts**

1. Hello, this is Sato. Nice to meet you.
2. (In case you do not know the subject’s name) Can I ask your name?
3. I live in Kyoto. It is very cold/cold/warm/very warm here today. How about over there?
4. Which do you like better, cold days or hot days?
5. So, please tell me about yourself.
6. What is your favorite food?
   - Why do you like it?
   - What food do you dislike?
   - Why do you dislike it?
7. What do you usually do at school?
   - What are you doing in special class?
   - Why did you select this class?
   - What other classes could you select?
8. Did you go on a school trip last year? What did you do during the school trip?
   - What was your aim on the school trip?
   - What were some good or bad memories from the school trip?
9. What do you want to do after graduation?
   - Why do you want to do it?
   - Do you have an actual plan for doing it?
10. What do you value?
    - How long has this been valuable to you?
11. Do you keep anything secret from your teachers?
    - Why do you keep it secret?

*The interlocutor asked the following questions of subjects when she had finished asking the questions listed above.*

1. Do you play video games?
   - What is your favorite game?
2. Do you like singing karaoke?
   - What do you sing?
3. I found a class named “FUREAI IGO” on the website of your school. Is it different from the game of Go (IGO)? Do you like it?
4. Could you tell me about Mr. XXX (teacher’s name)?
5. What do you do on your days off?
   - Why do you do it?
